# Heterogeneous structures formed by conserved RNA sequences within the HIV reverse transcription initiation site

**DOI:** 10.1261/rna.056804.116

**Published:** 2016-11

**Authors:** Aaron Coey, Kevin Larsen, Joseph D. Puglisi, Elisabetta Viani Puglisi

**Affiliations:** 1 Department of Structural Biology, Stanford University School of Medicine, Stanford, California 94305-5126, USA; 2 Biophysics Program, Stanford University School of Medicine, Stanford, California 94305-5126, USA

**Keywords:** HIV RNA structure, reverse transcription, NMR spectroscopy, single-molecule FRET

## Abstract

Reverse transcription is a key process in the early steps of HIV infection. This process initiates within a specific complex formed by the 5′ UTR of the HIV genomic RNA (vRNA) and a host primer tRNA^Lys^_3_. Using nuclear magnetic resonance (NMR) spectroscopy and single-molecule fluorescence spectroscopy, we detect two distinct conformers adopted by the tRNA/vRNA initiation complex. We directly show that an interaction between the conserved 8-nucleotide viral RNA primer activation signal (PAS) and the primer tRNA occurs in one of these conformers. This intermolecular PAS interaction likely induces strain on a vRNA intramolecular helix, which must be broken for reverse transcription to initiate. We propose a mechanism by which this vRNA/tRNA conformer relieves the kinetic block formed by the vRNA intramolecular helix to initiate reverse transcription.

## INTRODUCTION

**FIGURE 1.**
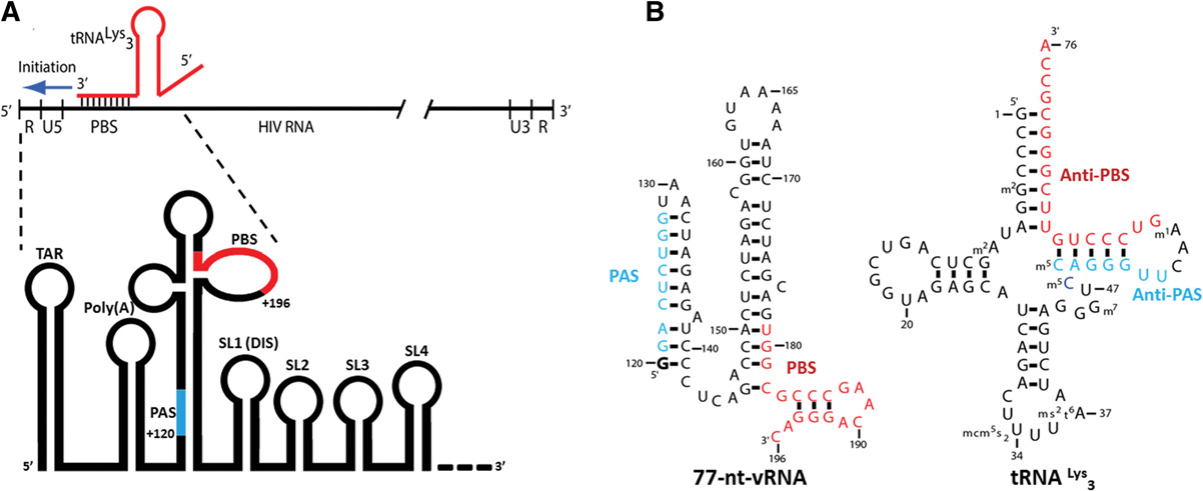
(*A*) The primer tRNA^Lys^_3_ anneals to the vRNA at the PBS site to form an 18-bp helix from which reverse transcription initiates. This site is found within the highly structured 5′ UTR. The proximal locations of the conserved vRNA PBS and PAS sequences are highlighted in red and blue, respectively. (*B*) The sequences of the 77-nt-vRNA construct and tRNA^Lys^_3_ primer contain the complementary PBS/anti-PBS and PAS/anti-PAS sequences highlighted in red and blue, respectively.

Reverse transcription is one of the first steps in HIV infection after the viral envelope fuses with the host cell membrane and the viral capsid enters the cell cytoplasm. Within the capsid are two copies of the single-stranded positive-sense viral RNA genome (vRNA) that must be reverse transcribed into double-stranded linear DNA, which is subsequently transported into the nucleus and integrated into the host cell genome. Reverse transcription is catalyzed by the viral enzyme reverse transcriptase (RT) and starts at the 3′ hydroxyl of a primer tRNA^Lys^_3_ that was prepackaged and preannealed from the previous host cell (Fig. 1A; Baltimore 1970; Temin and Mizutani 1970; Haseltine et al. 1976). The initiation of reverse transcription, during which the first approximately 15 dNTPs are incorporated onto the primer tRNA, is slow and nonprocessive relative to the subsequent elongation phase that proceeds toward the vRNA 5′ end (Pop and Biebricher 1996; Suo and Johnson 1997a,b; Liang et al. 1998; Goldschmidt et al. 2002). Such a slow initiation phase likely plays an important role in regulating the timing of reverse transcription initiation.

The binary complex formed between HIV genomic RNA and the annealed tRNA^Lys^_3_ is recognized by RT to initiate replication of the viral genome. Genetic, biochemical, and structural investigations have suggested a conserved RNA structure that is recognized by RT, but models are conflicting (Isel et al. 1995, 1998, 1999b; Pop and Biebricher 1996; Suo and Johnson 1997a,b; Liang et al. 1998; Lanchy et al. 2000; Goldschmidt et al. 2003, 2004; Tisne et al. 2004; Puglisi and Puglisi 2011; Seif et al. 2015). Three key regions of the RNA–RNA interaction have been proposed to guide the formation of the initiation complex: the primer-binding site (PBS), A-rich loop (ARL), and primer activation site (PAS) interactions. The roles of these RNA-pairing interactions in regulating initiation kinetics are supported by mutagenesis and biochemical experiments (Aiyar et al. 1992; Essink et al. 1995; Puglisi and Puglisi 1998; Beerens and Berkhout 2002a,b; Rigourd et al. 2003; Abbink et al. 2004; Ooms et al. 2007; Beerens et al. 2013; Jones et al. 2013; Sleiman et al. 2013; Seif et al. 2015).

**FIGURE 2.**
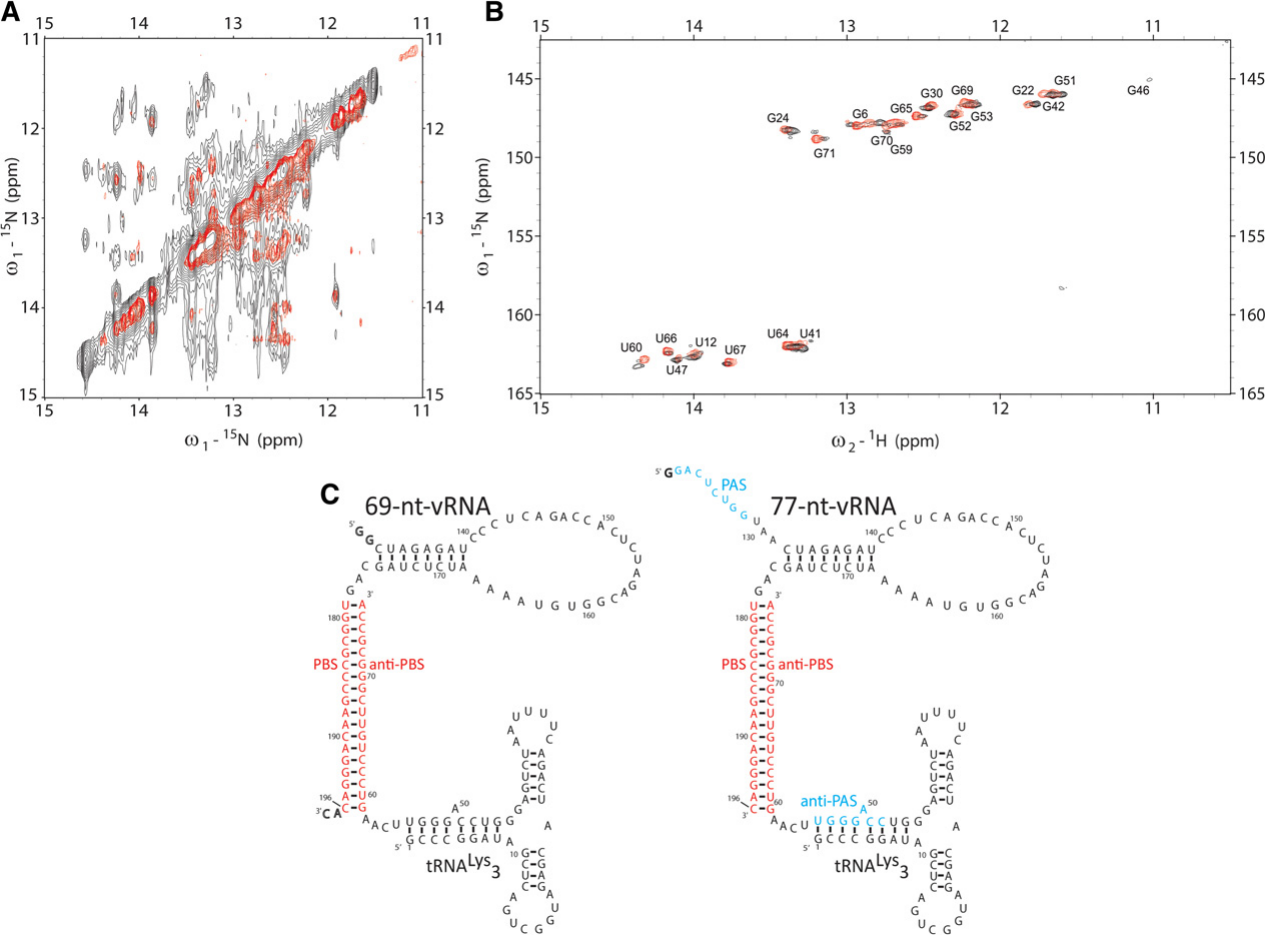
(*A*) The homonuclear spectrum of the 69-nt-vRNA/tRNA complex (red) overlaid on the 77-nt-vRNA/tRNA spectrum (black) shows that the 77-nt-vRNA/tRNA complex adopts a fold similar to the 69-nt-vRNA/tRNA spectrum. Several new NOEs within the 77-nt-vRNA/tRNA spectrum do not overlap with the 69-nt-vRNA/tRNA spectrum. (*B*) The ^15^N-^1^H TROSY spectrum of the 69-nt-vRNA/tRNA complex containing ^15^N-labeled tRNA (red) overlaid on the 77-nt-vRNA/tRNA ^15^N-^1^H TROSY spectrum containing ^15^N-labeled tRNA (black) shows that the tRNA adopts a similar fold when in complex with either 69-nt-vRNA or 77-nt-VRNA. (*C*) Secondary structure of the 77-nt-vRNA/tRNA complex showing a similar fold to the 69-nt-vRNA/tRNA complex.

The vRNA PBS is a sequence of 18 nucleotides (nt) that is complementary to the 3′ end of the primer tRNA^Lys^_3_ (Fig. 1B). This sequence is crucial for efficient tRNA priming and reverse transcription initiation*.* Upon annealing to the tRNA anti-PBS sequence to the vRNA PBS sequence, an 18-bp intermolecular RNA helix is formed. This helix serves as the binding site of RT, likely by positioning the helix in the large cleft in RT between the p51/p66 subunits, and is thus the initiation site of reverse transcription. The use of a 3′ end of a host tRNA to hybridize to viral RNA is a general mechanism for retroviral reverse transcription initiation (Haseltine et al. 1976). Prior NMR studies of HIV initiation complexes confirmed the presence of this intermolecular pairing (Tisne et al. 2004; Puglisi and Puglisi 2011). The formation of the PBS helix leads to significant rearrangements in both the tRNA by disrupting the D- and T-stem–loops and in the vRNA by forming an intramolecular helix between vRNA nucleotides 132–139 and 168–175 (Fig. 2C). However, our NMR studies were on constructs that did not contain the PAS sequence, described below. The ARL in viral RNA is proposed to interact with the U-rich anticodon of tRNA^Lys^_3_, stabilizing the initiation complex (Puglisi and Puglisi 1998; Rigourd et al. 2003; Jones et al. 2013; Seif et al. 2015).

The HIV PAS sequence is located ∼45 nt in the 5′ direction from the PBS (Fig. 1B; Beerens et al. 2001). This sequence is 8 nt in length and is complementary to an 8-nt anti-PAS sequence within the primer tRNA^Lys^_3_. A thorough phylogenetic study predicted that all known retroviruses have a PAS sequence that is complementary to its host tRNA primer (Beerens and Berkhout 2002a). In vitro mutational analysis of the PAS sequence in HIV demonstrated that this sequence is important for regulating reverse transcription initiation kinetics and that compensatory mutations of the PAS and PBS sequences can rescue defects in HIV replication when attempting to switch the priming tRNA to a different tRNA (Beerens and Berkhout 2002a). In vivo co-mutation of both the PAS and the PBS sequences also facilitates primer tRNA switching (Abbink et al. 2004). While these results highlight the importance of the PAS interaction in initiation and suggest a role during reverse transcription (Beerens et al. 2001), they do not delineate its precise role or point of formation in the initiation process.

Based on these data, the initiation complex is highly structured, but models for its structure are conflicting. Deleting regions of vRNA structure involved in reverse transcription initiation decreased virus viability in vivo and led to a high reversion rate to the wild-type sequence (Abbink et al. 2004). However, when RNA interactions form during the process of initiation remains unclear. The RNA interactions described may form simultaneously or in different functional states of the initiation complex. RNAs often adopt multiple conformers of similar stability, separated by relatively high-energy barriers for rearrangement, and proteins and ligands can modulate these equilibria.

To probe the dynamic conformational landscape of the HIV initiation complex, we have applied here two distinct biophysical techniques that embrace conformational exchange—NMR and single-molecule fluorescence spectroscopy. NMR spectroscopy probes RNA secondary and tertiary structures; through analysis of exchangeable imino proton (^1^H) NMR spectra, the base pairing of an RNA can be rapidly obtained. Distinct conformations in slow (on NMR time scale, lifetimes > 100 msec) exchange are revealed as separate resonances, allowing conformational exchanges to be probed at the single-nucleotide level. Single-molecule fluorescence resonance energy transfer (smFRET) spectroscopy provides direct information on conformation as a function of time; RNAs are labeled with fluorescent donor and acceptor dyes, and single molecules of the doubly labeled RNAs are monitored continuously upon donor excitation as a function of time. Dipolar energy transfer to the acceptor dye depends on 1/*R*^6^, where *R* is the interdye distance.

These methods were applied to the binary HIV initiation complex that recapitulates the potential PBS, ARL, and PAS interactions between the vRNA and tRNA. A 100-nt region of vRNA (Lu et al. 2011) is sufficient to recapitulate the major RNA elements responsible for regulating reverse transcription initiation; mutating sequences outside this region does not affect initiation kinetics (Isel et al. 1998). Guided by prior biochemical studies, we could minimize the key region of vRNA to 77 nt (Fig. 1B; Puglisi and Puglisi 2011). Using NMR, we demonstrate that the 77-nt vRNA in complex with the primer tRNA adopts two conformations and assign a full secondary structure to each of these conformations. We employ smFRET to confirm the presence of the two conformations in slow exchange on a minutes timescale and modulate the population distributions by mutating the PAS sequence. Our results highlight the role of RNA conformational equilibria in HIV RT initiation and underscore the power of complementary NMR and smFRET methods to delineate the dynamic behavior of heterogeneous RNA structures.

## RESULTS

### Purification of a 1:1 77-nt-vRNA/tRNA complex

To understand the interactions of the vRNA reverse transcription initiation site with the primer tRNA^Lys^_3_, we focused on a region of 100 nt between residues 123 and 217 in the HIV-1 Mal isolate previously shown to form a complex with tRNA^Lys^_3_, recapitulating the biochemical properties of complexes formed with full-length viral RNAs (Isel et al. 1998). Previously, we investigated both a 99- and 69-nt fragment from this region of the Mal isolate; the 99mer was poorly behaved for structural studies, but the 69mer was extensively investigated using biochemical and NMR methods (Puglisi and Puglisi 2011). The 69-nt sequence from this region was capable of annealing to tRNA^Lys^_3_ and formed a specific complex with a well-defined secondary structure as determined by NMR (Fig. 2C). Upon formation of a 1:1 tRNA–vRNA complex using the 69-nt sequence, the tRNA T-stem and acceptor stem completely unfold to anneal the tRNA anti-PBS sequence to the vRNA PBS. The tRNA D-stem and anticodon-stem are preserved, and the vRNA forms an intramolecular helix between complementary regions downstream from the PBS. However, this construct did not contain the 8-nt PAS sequence, which is immediately beyond the 5′ end of the 69mer and is complementary to a sequence in the tRNA T-stem. To investigate how the PAS-anti-PAS interaction might change the initiation complex, we designed a 77-nt-vRNA from the HIV-1 MAL sequence that extended the 69-nt construct to include the conserved PAS sequence for direct study by NMR spectroscopy (Fig. 1B).

To probe the role of the PAS interaction in the initiation complex, we formed a 1:1 vRNA/tRNA complex between in vitro transcribed 77-nt-vRNA and full-length tRNA^Lys^_3_ using heat annealing. This method has been shown to produce vRNA/tRNA complexes similar to those formed through nucleocapsid-mediated annealing (Brule et al. 2002; Iwatani et al. 2003; Sleiman et al. 2012; Beerens et al. 2013). The resulting heat-annealed reaction yielded several higher order species upon native PAGE analysis. These species were eliminated by size exclusion chromatography to produce a pure 1:1 vRNA/tRNA complex (Supplemental Figs. S1, S2). This 1:1 complex was stable at room temperature for days and slowly formed higher order complexes only at high concentrations (500 µM) on a timescale (days) longer than the performed NMR experiments.

### Initial NMR characterization

The HIV 77-mer-tRNA^Lys^_3_ complex was first investigated using homonuclear ^1^H NMR spectroscopy. To observe directly if the 77-nt-vRNA construct forms a complex with the tRNA similar to the complex formed by the previous 69-nt construct, we prepared an unlabeled complex for homonuclear NMR experiments. The imino ^1^H 1D spectrum of the 77-mer-tRNA^Lys^_3_ complex was nearly similar to that previously obtained on the 69-mer-tRNA^Lys^_3_ complex, suggesting similar secondary structures (Fig. 2A). The secondary structure of the 77-mer-tRNA complex was probed using a NOESY experiment to detect imino–imino ^1^H NOEs for adjacent base pairs in an RNA helix. The resulting spectrum showed significant overlap of NOE crosspeaks due to the spectral complexity and linewidth of imino ^1^H resonances from the size of this complex (50 kDa). Despite this spectral overlap, overlaying the homonuclear NOESY spectrum of the 69-nt-vRNA/tRNA complex onto the 77-nt-vRNA/tRNA complex revealed that all imino–imino ^1^H NOE crosspeaks in the previous 69-nt-vRNA/tRNA are also present in the 77-nt-vRNA/tRNA complex (Fig. 2A).

To confirm that similar structures are formed, we prepared a 77-nt-vRNA/tRNA complex with ^15^N-labeled tRNA. Chemical shifts of both ^1^H and ^15^N are highly sensitive to the local environment, providing a rapid qualitative probe of macromolecular structure. The ^15^N-^1^H TROSY spectrum of the ^15^N-tRNA-77-mer vRNA was superimposable on that of an ^15^N-labeled tRNA-69-mer vRNA complex, strongly supporting that tRNA^Lys^_3_ adopts a similar fold in the two complexes in which the D-stem and anticodon stem are preserved and the tRNA anti-PAS sequence is annealed to the 5′ end of the tRNA (Fig. 2B). Many RNAs, including tRNA^Lys^_3_, require Mg^2+^ ions to fold into their proper tertiary structure. We probed the Mg^2+^ dependence of the 77-mer-tRNA by acquiring NMR spectra at a range of Mg^2+^ concentrations to 10 mM Mg^2+^ and observed no change in the 1D ^1^H NMR spectra over this range (Supplemental Fig. S3). Based on this comparison, we concluded that the 77-nt-vRNA/tRNA complex adopts an overall fold similar to the 69-nt-vRNA/tRNA complex and that the conformation adopted is independent of Mg^2+^ concentration.

### Assigning the intermolecular PAS interaction

**FIGURE 3.**
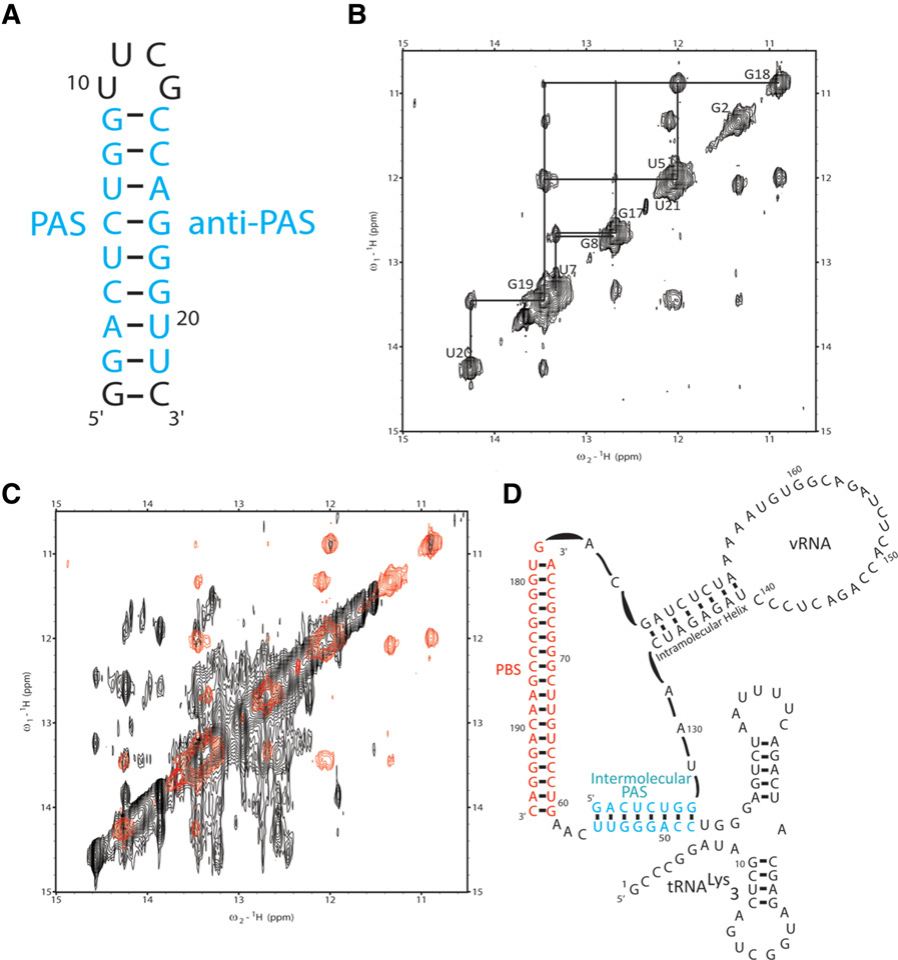
(*A*) The 5′ side of the PAS oligo was constructed from the vRNA PAS sequence and the 3′ side from the tRNA anti-PAS sequence. The stem is capped by a UUCG tetraloop on one end and a GC base pair on the other. (*B*) Assigning the homonuclear NOESY spectrum of the PAS oligo shows that it folds into a single stem–loop structure. (*C*) Overlaying the PAS oligo NOESY spectrum (red) onto the 77-nt-vRNA/tRNA NOESY spectrum (black) shows that the 77-nt-vRNA/tRNA complex contains a PAS/anti-PAS secondary structural interaction. (*D*) NOESY data show that the vRNA PAS sequence displaces the tRNA 5′ end to base pair with the tRNA anti-PAS sequence.

Overlaying the NOESY spectra of the 69-nt-vRNA/tRNA and 77-nt-vRNA/tRNA complexes also allowed us to identify several imino–imino NOEs in the larger complex that could not be assigned (Fig. 2A). We hypothesized that these NOEs were the result of the formation of a new secondary structure within the complex, which could potentially be an intermolecular PAS interaction between the vRNA PAS and tRNA anti-PAS sequences. NMR spectroscopic work was previously performed on oligonucleotides annealed to the tRNA to mimic an intermolecular PAS interaction, but these experiments were not performed in the context of longer tRNAs (Sleiman et al. 2013). To assign unambiguously the additional peaks observed in the larger 77-nt-vRNA/tRNA complex, we first designed a small 22-nt RNA to mimic the secondary structure formed by an intermolecular vRNA/tRNA PAS interaction (Fig. 3A). The 5′ end of this RNA oligonucleotide contains the 8-nt vRNA PAS sequence, and the 3′ end contains the complementary tRNA anti-PAS sequence. The two sequences are oriented such that they will base pair with one another capped by a -UUCG- tetraloop at one end and an additional GC base pair at the other. We investigated the conformation of this 22-nt oligonucleotide by NMR.

The homonuclear NOESY spectrum of the 22-nt oligo displayed sharp, well-dispersed imino NOE resonances that were readily assigned and attributed to the formation of a single stem–loop structure (Fig. 3B). We overlaid this assigned spectrum with the full-length 77-nt-vRNA/tRNA NOESY spectrum and observed that five of the imino resonances found within the center of the 22-nt oligo stem overlapped with previously unassignable resonances in the 77-nt-vRNA/tRNA NOESY spectrum (Fig. 3C). Of particular importance is the unique wobble U5-G18 base pair. Resonances from G–U pairs are normally observed farther upfield in the spectrum compared to the Watson-Crick G-C and A-U base pairs and serve as excellent starting points for secondary structural assignments by NMR. A set of resonances from this G-U pair is observed in the 77mer at similar chemical shifts as observed in the 22-mer oligonucleotide (Fig. 3C) and was key in determining the presence of an intermolecular vRNA/tRNA PAS interaction. From these data, we concluded that the 77-nt-vRNA/tRNA complex exhibits an intermolecular vRNA/tRNA interaction whereby the vRNA PAS sequence anneals to the tRNA anti-PAS sequence (Fig. 3D). We attributed the inability to observe imino proton peaks for the bases at the terminal ends of the PAS interaction for the 77-nt-vRNA/tRNA complex to differences in magnetic environment that the protons would experience in the context of the 22-nt oligo and full-length complex and potential exchange due to fraying of the terminal pairs in the larger RNA.

### Identifying two RNA conformers

The NMR data presented above present a conflicting view of the initiation complex conformation. To recapitulate NOE assignments from the 69-nt-vRNA/tRNA and 22-nt oligo PAS-mimetic within the same NOESY spectrum of the 77-nt-vRNA/tRNA spectrum suggests the simultaneous presence of an intermolecular PAS interaction between the vRNA and tRNA and an intramolecular anti-PAS interaction where the tRNA 5′ end is annealed to the tRNA anti-PAS sequence. A viable explanation for the observed data is the presence of two conformers formed by the vRNA/tRNA complex in slow exchange on an NMR timescale (exchange lifetime time >100 msec). The first of these conformers forms an intermolecular PAS interaction whereby the vRNA PAS sequence anneals to the tRNA anti-PAS sequence, and the second conformer forms an intramolecular anti-PAS interaction in which the tRNA 5′ end anneals to the tRNA anti-PAS sequence to sequester it from the vRNA.

We used smFRET spectroscopy to probe whether a heterogeneous population of conformers was adopted by the HIV 77-mer-tRNA^Lys^_3_ complex. smFRET can parse the structural and dynamic properties of heterogeneous conformer populations when bulk techniques such as NMR yield mixed results. In a FRET experiment, a donor dye is electronically excited via laser. In the absence of an acceptor dye, the donor dye will fluoresce and relax back to the electronic ground state. If an acceptor dye with an absorbance spectrum that overlaps the emission spectrum of the donor dye is in close proximity (<80 Å) to the donor dye, energy can be transferred from the donor dye to the acceptor dye through dipole–dipole interactions, leading to acceptor dye emission; the efficiency of this transfer is proportional to 1/*r*^6^, where *r* is the interdye distance. By observing FRET from a collection of single RNA molecules labeled at appropriate positions with donor and acceptor dyes, the conformational dynamics of the RNA can be delineated directly (Zhuang et al. 2000; Russell et al. 2002; Liu et al. 2005; Solomatin et al. 2010; Beerens et al. 2013).

**FIGURE 4.**
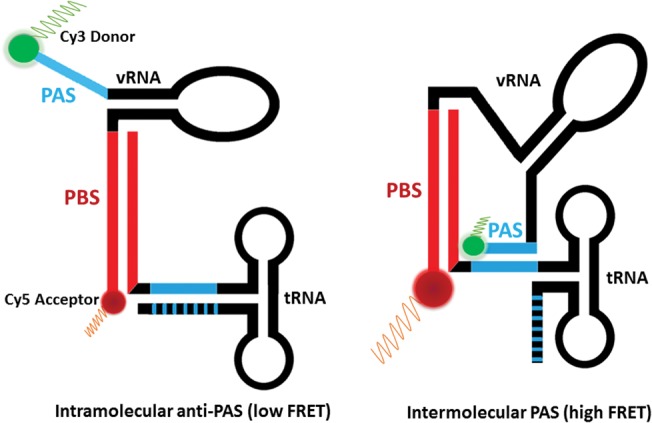
Placing donor and acceptor dyes at the 5′ and 3′ ends of the 77-nt-vRNA, respectively, allows the direct monitoring of PAS/anti-PAS interactions. A low FRET signal indicates that the PAS/anti-PAS interaction is not present, whereas a high FRET signal indicates that the PAS/anti-PAS sequences have annealed.

We designed a labeling scheme for the 77-mer-tRNA^Lys^_3_ complex that directly tested the presence of the potential PAS interactions. We placed Cy3 donor and Cy5 acceptor dyes at the 5′ and 3′ ends of the 77-nt-vRNA, respectively, and annealed this construct to a biotinylated tRNA^Lys^_3_ for smFRET measurements (Fig. 4). This labeling scheme allows direct observation of the intermolecular PAS interaction through FRET measurements on surface-immobilized initiation complexes for up to 10 min. Interdye distances were modeled in the PyMOL Molecular Graphics System, Version 1.8 Schrödinger, LLC and Rosetta using ideal RNA A-form helix geometries (Das et al. 2010). If an intermolecular vRNA/tRNA PAS interaction forms, the two dyes will be in close proximity (<20 Å) and have a high-energy transfer efficiency (high FRET). If the intramolecular anti-PAS interaction occurs, the donor Cy3 dye will be occluded from the tRNA anti-PAS site and is at a greater distance (>40 Å) from the acceptor Cy5 dye leading to less efficient energy transfer (low FRET). Previous work used smFRET to analyze structural rearrangements within the initiation complex; however, this labeling scheme did not allow the direct observation of the intermolecular PAS interaction (Beerens et al. 2013).

**FIGURE 5.**
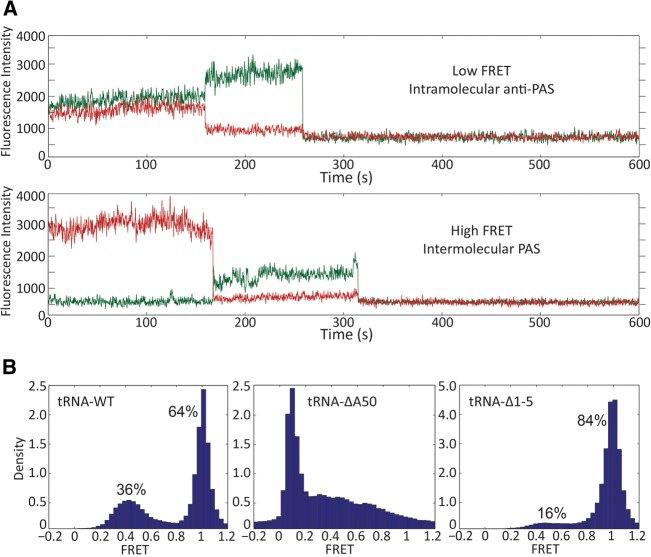
(*A*) Two distinct FRET states were observed for the wild-type 77-nt-vRNA/tRNA FRET complex. The low FRET state (*above*) at 0.4 FRET efficiency was attributed to the intramolecular anti-PAS conformation, and the state (*below*) at 1.0 FRET efficiency was attributed to the intermolecular PAS conformation. (*B*) Approximately one-third of the wild-type complexes is in the low FRET intramolecular anti-PAS conformation while two-thirds are in the high FRET intermolecular PAS conformation (*n* = 167). The tRNA-ΔA50 mutation completely ablates the high FRET state (*n* = 172), and the tRNA-Δ1-5 mutation shifts the majority of the complexes to the high FRET state (*n* = 233).

smFRET experiments showed that the 77-nt-vRNA/tRNA complex exhibits two distinct FRET states (Fig. 5A). We assigned the high FRET state (1.0 FRET efficiency) to the intermolecular PAS interaction and the low FRET state (0.4 FRET efficiency) to the intramolecular anti-PAS interaction. Quantifying the time each molecule spends in the high and low FRET states showed that 64% of molecules are in an intermolecular PAS conformation and 36% are in an intramolecular anti-PAS conformation (Fig. 5B). This population distribution is independent of Mg^2+^ (Supplemental Table S1), and the conformers do not interconvert on a timescale measureable before dye photobleaching (>10 min).

To test the assignment of these FRET states to the intra- and intermolecular PAS interaction, we perturbed the conformational equilibrium of the populations through tRNA mutation. The first mutation we made, tRNA-ΔA50, deletes the tRNA adenine at nucleotide position 50. In an intramolecular anti-PAS conformation, this mutation deletes the bulged A50 nucleotide to stabilize the tRNA anti-PAS helix and increases the energy barrier for forming the intermolecular PAS interaction by requiring a bulge to form on the vRNA PAS to anneal to the tRNA. Quantifying the FRET distributions showed that this point mutation completely ablates the intermolecular PAS high FRET state (Fig. 5B).

To favor the intermolecular PAS interaction and thus increase the population of high FRET conformers, we deleted the first five nucleotides of the 5′ end of the tRNA to form a mutant that we named tRNA-Δ1-5. This deletion mutation partially exposes the tRNA anti-PAS sequence by removing 5′ tRNA nucleotides, which normally participate in the intramolecular anti-PAS interaction. The guanine at tRNA position 6 was retained for in vitro transcription efficiency for NMR experiments. The FRET distribution for the tRNA-Δ1-5 complex showed an increase in the high FRET population to 84% and a decrease in the low FRET population to 16%, demonstrating that the 5′ end of the tRNA is responsible for occluding the vRNA PAS sequence from annealing in an intermolecular PAS conformation (Fig. 5B).

### Determining intermolecular PAS complex secondary structure

We were confident in our secondary structural assignment for the intramolecular anti-PAS conformer because of its similarity to the previously studied 69-nt-vRNA/tRNA complex and sought to determine the secondary structure of the intermolecular PAS complex. We performed structural modeling of the intermolecular PAS conformer in PyMol and Rosetta to model whether the vRNA intramolecular helix observed in the 69-nt-vRNA/tRNA complex is a viable structure upon vRNA PAS binding to the tRNA anti-PAS. In silico modeling demonstrated that the PBS helix, vRNA intramolecular helix, and PAS interaction can simultaneously be present without breaking covalent bonds.

**FIGURE 6.**
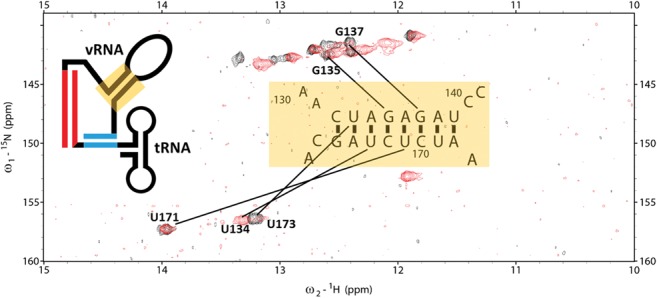
The ^15^N-^1^H TROSY spectrum of ^15^N-labeled 77-nt-vRNA in complex with unlabeled tRNA-ΔA50 (red) overlaid onto the ^15^N-^1^H TROSY spectrum of ^15^N-labeled 69-nt-vRNA in complex with unlabeled wild-type tRNA (black) shows that the formation of the intermolecular PAS interaction maintains the vRNA intramolecular helix. Resonances belonging to imino protons in the vRNA intramolecular helix are labeled.

To confirm directly that the vRNA intramolecular helix is maintained upon formation of the intermolecular PAS interaction, we isotopically ^15^N-labeled the vRNA and annealed it to the tRNA-Δ1-5 mutant. smFRET experiments showed that this mutant primarily adopts the intermolecular PAS conformation. The small population of low FRET conformers (16%) is below the NMR detection threshold, and only intermolecular PAS conformers would be observed. We used this mutant as a model to directly observe the secondary structures formed by the vRNA upon intermolecular PAS annealing. ^15^N-^1^H TROSY experiments showed at least five resonances assigned to the vRNA intramolecular helix are preserved and have NOE cross peaks upon vRNA PAS annealing and that the PBS helix is maintained (Fig. 6). From these data, we concluded that the intermolecular PAS conformer exhibits the PBS, PAS, and vRNA intramolecular helices.

## DISCUSSION

Previous attempts to define the secondary structure of the HIV reverse transcription initiation site have led to conflicting models. These results are likely a product of the many methods (chemical modification, enzymatic digests, FRET, and NMR) used to determine structural models and the variation in construct lengths and HIV strains (Isel et al. 1995, 1999a; Suo and Johnson 1997a; Beerens et al. 2001; Brule et al. 2002; Goldschmidt et al. 2003, 2004). In this work, we used NMR spectroscopy to assign unambiguously RNA secondary structures to a 77-nt vRNA containing the conserved PAS sequence in complex with tRNA^Lys^_3_ by directly observing imino protons participating in RNA base pairs. Our vRNA/tRNA complex is ∼50 kDa in size and is structurally heterogeneous. Using smFRET spectroscopy as a complementary technique, we showed that two conformations exchange slowly on a minutes timescale, with two-thirds of the complexes in an intermolecular PAS conformation and the other third in an intramolecular anti-PAS conformation. We used smFRET data to design vRNA/tRNA complex mutants that favored one of the two conformations and aided in assigning a full secondary structure to both conformations.

In the intramolecular anti-PAS conformation, the viral PAS sequence is not involved in secondary structure. In this conformation, the 18-nt vRNA PBS sequence anneals to the complementary tRNA anti-PBS sequence to form the RNA/RNA helix where RT binds and reverse transcription initiates. To form the PBS helix, the tRNA T-stem and acceptor stem unfold, and the 5′ tRNA end anneals to a second internally complementary sequence. The D-stem and anticodon stem are preserved. The vRNA forms an internal intramolecular helix 3 nt upstream of the PBS helix (Fig. 2C).

Subsequent NMR experiments on the mutant 77-nt-vRNA/tRNA-Δ1-5 complex revealed the formation of an intermolecular PAS conformation in which the vRNA PAS sequence is annealed to the tRNA anti-PAS sequence and the 5′ end of the tRNA is displaced. The vRNA intramolecular helix, PBS helix, and tRNA D-stem and anticodon loops structures are still preserved (Fig. 3D). Chemical mapping and mutational functional studies previously identified a potential interaction between an ARL in the vRNA between nucleotides 164 and 168 and the tRNA anticodon loop (Puglisi and Puglisi 1998, 2011; Beerens and Berkhout 2002b; Rigourd et al. 2003; Tisne et al. 2004; Jones et al. 2013). While some work suggested that the 2-thio-uridine modification at tRNA nucleotide 34 potentially stabilizes this interaction, we observed evidence through chemical mapping that such an interaction occurred in unmodified, in vitro*-*transcribed tRNA (Puglisi and Puglisi 2011). We did not find evidence for such an interaction in any of our NMR spectra, indicating that this interaction does not occur or is too shortly lived to observe on an NMR timescale in these complexes.

Both the intramolecular and intermolecular PAS conformations are formed by the wild-type HIV sequence and exist simultaneously. The exchange between the two conformations is slow (minutes timescale) and limited by our observation of the fluorescent dyes before photobleaching. Individual RNA molecules exchange rarely between the two conformational states for the wild-type sequence, demonstrating that there exists a high-energy barrier for rearrangement of the RNA conformation. Prior single-molecule FRET studies on RT-RNA dynamics, using FRET between RT and RNA (Abbondanzieri et al. 2008; Liu et al. 2008, 2010), have highlighted this conformational heterogeneity as well.

Our results show unambiguously that the HIV initiation complex exists in two conformational states, reconciling conflicting results and models of the initiation complex. Conserved RNA sequences in this region can adopt multiple conformations with vRNA sequences as small as 77 nt. The majority of prior structural work on this system was done through chemical mapping experiments that are not easily amenable to parsing multiple conformations within a bulk sample. These experiments were also performed on vRNAs ranging up to 300 nt in length from distinct HIV strains, which could potentially adopt more than just the two conformations observed in this study.

The best structurally characterized HIV-1 strains are currently the HXB2, NL4.3, and Mal strains. These strains differ slightly in the 77-nt region we studied, but all contain PAS sequences. The existence of a physical intermolecular PAS interaction within each of these strains has been previously questioned with two studies concluding that this interaction only occurs in the Mal strain (Goldschmidt et al. 2003, 2004). For this reason, we focused our work on the HIV-1 Mal strain. We directly observed such an intermolecular PAS interaction within this strain but also demonstrated that not all complexes in this strain adopt this conformation. These data suggest that the PAS interaction may also occur within the HXB2 and NL4.3 strains but may be transient and not easily detected by previously used methods.

The two conformers observed within the 77-nt-vRNA/tRNA complex may be responsible for regulating reverse transcription initiation. When RT initiates reverse transcription, there are several pause sites of elongation that decrease RT processivity. One prominent pause site is located 3 nt downstream from the initiation site where the vRNA intramolecular helix forms (Liang et al. 1998; Goldschmidt et al. 2002). This helix induces RT pausing by providing an energy barrier that RT must overcome by melting the helix before proceeding. The helix also induces RT to adopt a reverse transcription incompetent conformation where it binds in a reverse mode to the PBS helix (Suo and Johnson 1997a,b). Our structural results suggest that the binding of the vRNA PAS sequence to the tRNA may serve to destabilize the vRNA intramolecular helix by inducing steric strain and thus reduce RT pausing. Such a mechanism may regulate when reverse transcription initiates through RNA structural control, as suggested previously (Ooms et al. 2007). Our results explain why mutations within the PAS sequence or PBS sequence alone interfere with initiation kinetics and why both sequences must be mutated to match the primer tRNA to rescue initiation kinetics when attempting to switch tRNA primers; a vRNA lacking a PAS sequence complementary to the tRNA anti-PAS sequence would be unable to anneal to the tRNA and facilitate the opening of the vRNA intramolecular helix. These hypotheses must be tested through further experiments.

Our results present a new perspective on RNA structures formed within the reverse transcription initiation complex in HIV. The ability to combine direct bulk NMR measurements with smFRET spectroscopy revealed two conformations adopted by the same sequences of RNA within the HIV reverse transcription initiation complex. These structures highlight the importance of considering biological regulation not in terms of one single structure but as multiple structures along a pathway to initiation. Further investigations of the role of RT in changing this RNA conformational landscape and how this process occurs in the context of full-length viral RNAs are required. Detailed investigations by NMR spectroscopy, X-ray crystallography, or cryo-electron microscopy will present deeper insights into the regulation of reverse transcription initiation by RNA structures in HIV.

## MATERIALS AND METHODS

### RNA preparation and complex formation

tRNA Lys3 and vRNA constructs were prepared by T7 polymerase in vitro transcription as previously described (Puglisi and Puglisi 2011). Elongated tRNA transcripts for smFRET experiments were transcribed with the addition of 1.25 µM biotin-GMP (TriLink) to the reaction mixture. Transcripts were denatured in 8 M urea and purified on a sequencing PAGE gel. Gel extraction was performed using 0.3 M ammonium acetate. Following ethanol precipitation, the RNA was dissolved in 10 mM bis-tris propane, pH 7.0, 10 mM NaCl for storage at −20°C. The PAS oligonucleotide was purchased from Integrated DNA Technologies.

vRNA/tRNA complexes were formed by mixing vRNA and tRNA in a 1:1 molar ratio at 1.5 µM each in 10 mM bis-tris propane, pH 7.0, 10 mM NaCl. The mixture was heated to 90°C then slowly cooled to room temperature over a period of 20 min. The NaCl concentration was increased to 50 mM for size exclusion FPLC. Heterodimeric vRNA/tRNA complex was purified from unannealed monomer and higher order species using a Superdex 200 (26/60) gel filtration column with 10 mM bis-tris propane, pH 7.0, 50 mM NaCl. The presence of a single dimeric species was confirmed by native PAGE, and the samples were concentrated on a Vivaspin 20 10,000 MWCO filter. Samples to be used for NMR spectroscopy were subsequently buffer exchanged into phosphate buffer.

### NMR spectroscopy

NMR spectra were collected at 25°C on a Varian INOVA 800 MHz NMR spectrometer equipped with CryoProbe at the Stanford Magnetic Resonance Laboratory. Samples were 250 µL in Shigemi NMR tubes, 300–600 µM in concentration, and buffered in 10 mM sodium phosphate, pH 6.5, 50 mM NaCl 90% H_2_O/10% D_2_O. Spectra were obtained using standard homo- and heteronuclear experiments optimized for RNA structure determination (RNAPack). Spectra were processed in VNMR, and ^1^H and ^15^N assignments were made in Sparky.

To assign the imino proton resonances, two-dimensional SSNOESY spectra were collected with 50, 100, and 200 msec mixing times for the vRNA/tRNA complex and the PAS oligo. SSNOESY experiments were obtained in 4–8 h. ^1^H-^15^N TROSY spectra were collected using T1-optimized experiments to improve the signal-to-noise ratio. The recycle delay was 0.5 sec, and spectra were collected in 8–12 h.

### smFRET experiments

Biotinylated tRNA for single-molecule experiments was produced by extending the 5′ end of the tRNA constructs to include a GG(UC)_10_ sequence. FPLC-purified vRNA/tRNA complexes for smFRET experiments were diluted to 200 pM and immobilized on a neutravidin-derivitazed quartz slide and washed with 10 mM bis-tris propane, pH 7.0, 50 mM NaCl. Immediately before collecting data, the slide was washed in the same buffer with the addition of an oxygen-scavenging system (2.5 mM 3,4 dihydroxybenzoic acid, 250 nM protocatechuate dioxygenase, 1 mM Trolox).

Single-molecule experiments were performed using a prism-based total internal reflection instrument as previously described (Marshall et al. 2008). smFRET measurements were performed using a diode-pumped solid-state 532 nm laser with 50 mW power measured at the prism. A Quad-View device (Photometrics) separated fluorescence emission into channels corresponding to Cy3 and Cy5 fluorescence. An EMCCD camera (Andor Technology) was used to record the fluorescence signal with an exposure of 100 msec per frame for 10 min. FRET traces were analyzed using home-written scripts in MATLAB (MathWorks). The population distribution between the high and low FRET states was calculated by fitting mixed Gaussian functions to the resulting FRET data.

## SUPPLEMENTAL MATERIAL

Supplemental material is available for this article.

## Supplementary Material

Supplemental Material
