# Supplementary material for: Heterogeneous structures formed by conserved RNA sequences within the HIV reverse transcription initiation site
Source: RNA. 2016 Nov;22(11):1689–98. doi: 10.1261/rna.056804.116 (PMC5066621; doi:10.1261/rna.056804.116)
Supplement: Supplemental Material [file supp_22_11_1689__index.html]

Heterogeneous structures formed by conserved RNA sequences within the HIV reverse transcription initiation site — Supplemental Material 

# Heterogeneous structures formed by conserved RNA sequences within the HIV reverse transcription initiation site

## Supplemental Material

- Supp\_FigS1.tif
- Supp\_TableS1\_Legends.docx
- Supp\_FigS2.tif
- Supp\_FigS3.tif
