# Supplementary figures and images for: Heterogeneous structures formed by conserved RNA sequences within the HIV reverse transcription initiation site
Source: RNA. 2016 Nov;22(11):1689–98. doi: 10.1261/rna.056804.116 (PMC5066621; doi:10.1261/rna.056804.116)

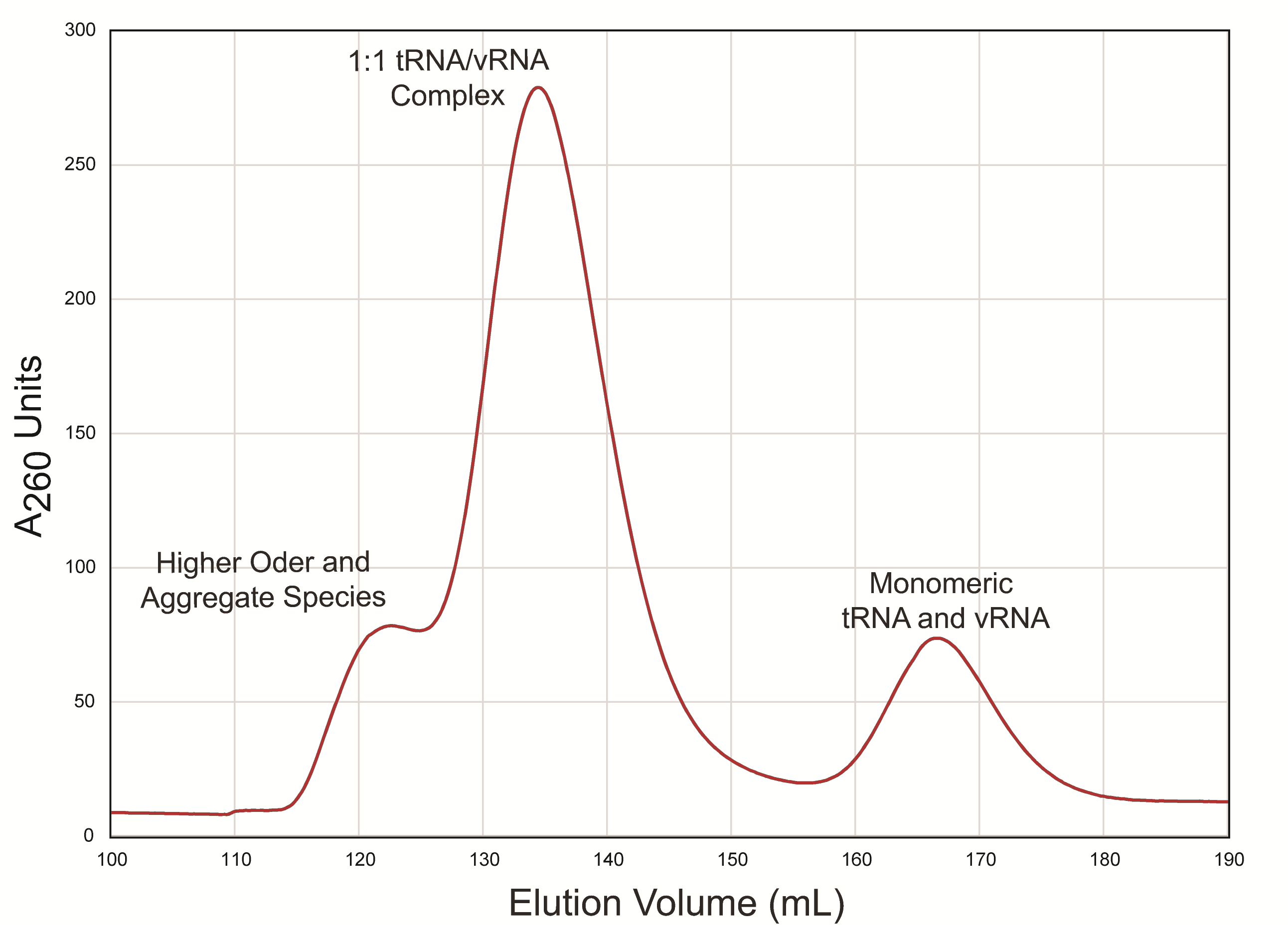

Supplement: Supplemental Material [file supp_056804.116_Supp_FigS1.tif]

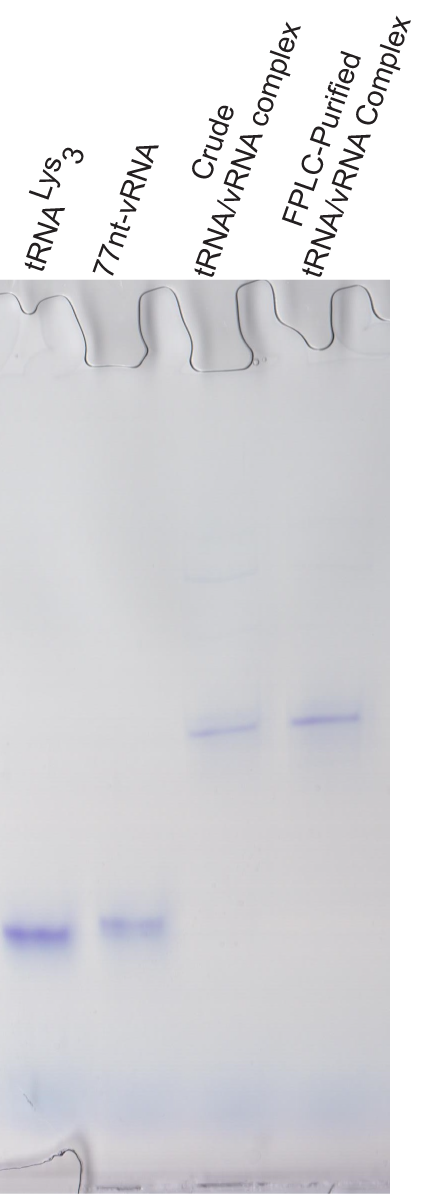

Supplement: Supplemental Material [file supp_056804.116_Supp_FigS2.tif]

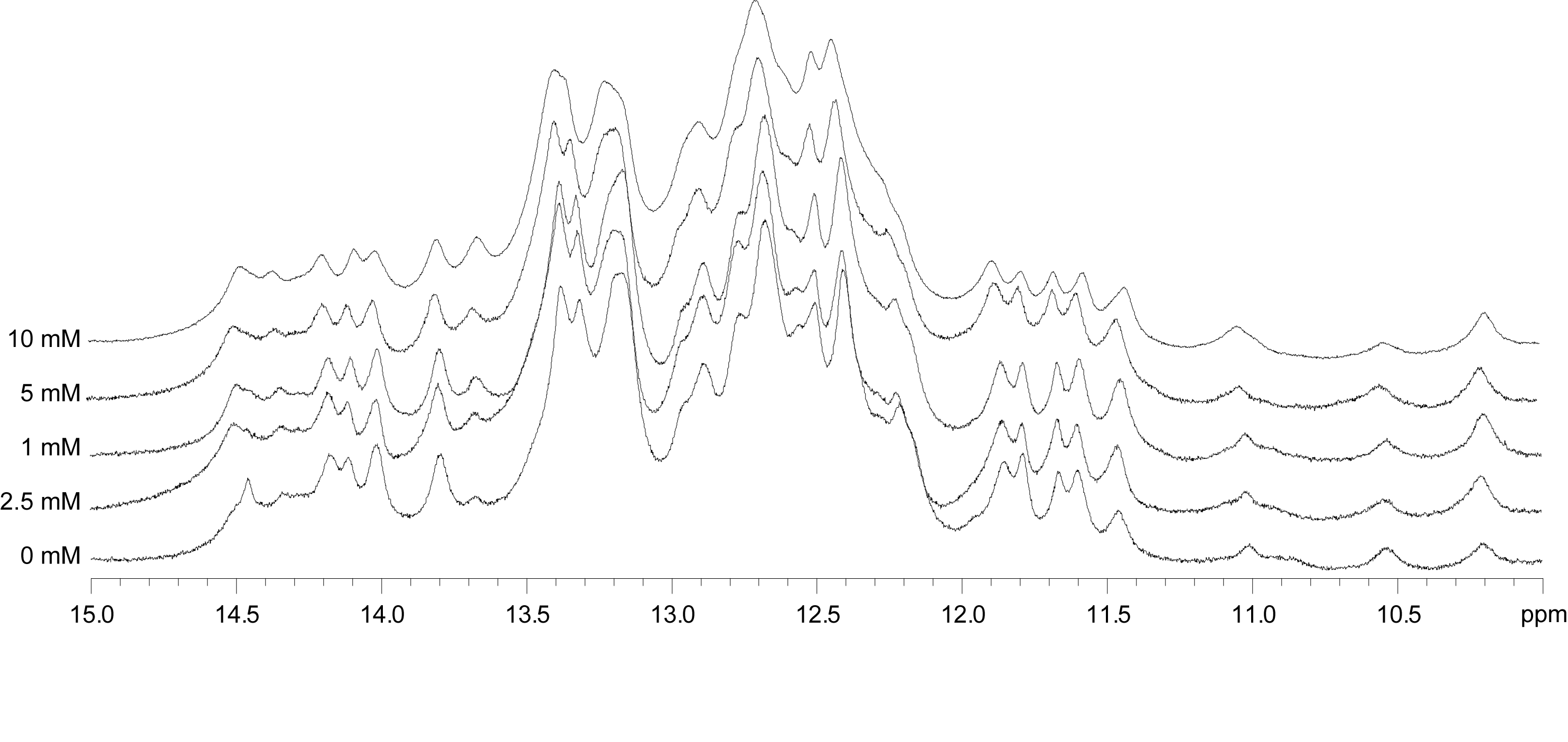

Supplement: Supplemental Material [file supp_056804.116_Supp_FigS3.tif]
