# Supplementary material for: Heterogeneous structures formed by conserved RNA sequences within the HIV reverse transcription initiation site
Source: RNA. 2016 Nov;22(11):1689–98. doi: 10.1261/rna.056804.116 (PMC5066621; doi:10.1261/rna.056804.116)
Supplement: Supplemental Material [file supp_056804.116_Supp_TableS1_Legends.docx]

**Table S1: Magnesium Titration into FRET Complex**

| **[MgCl_2_]** | **Low FRET** | **High FRET** |
| --- | --- | --- |
| 0 mM | 36% | 64% |
| 1 mM | 38% | 62% |
| 2.5 mM | 32% | 68% |
| 5 mM | 37% | 63% |
| 10 mM | 31% | 69% |

**Figure S1:** FPLC purification of the heat-annealed vRNA/tRNA complex separates impurities resulting from unannealed or higher order species from a 1:1 complex.

**Figure S2:** Natve PAGE shows that a pure 1:1 vRNA/tRNA complex can be obtained through heat annealing and subsequent size exclusion chromatography.

**Figure S3:** Mg^2+^ titration up to 10 mM does not affect the fold of the wild type 77-nt-vRNA/tRNA complex as determined by 1D ^1^H NMR.

**Table S1:** Mg2+ titration up to 10mM does not affect the conformational FRET distribution in smFRET experiments with the wild type 77-nt-vRNA/tRNA complex.
